# Supplementary figures and images for: TGF-β Pathways Stratify Colorectal Cancer into Two Subtypes with Distinct Cartilage Oligomeric Matrix Protein (COMP) Expression-Related Characteristics
Source: Biomolecules. 2022 Dec 14;12(12):1877. doi: 10.3390/biom12121877 (PMC9775768; doi:10.3390/biom12121877)

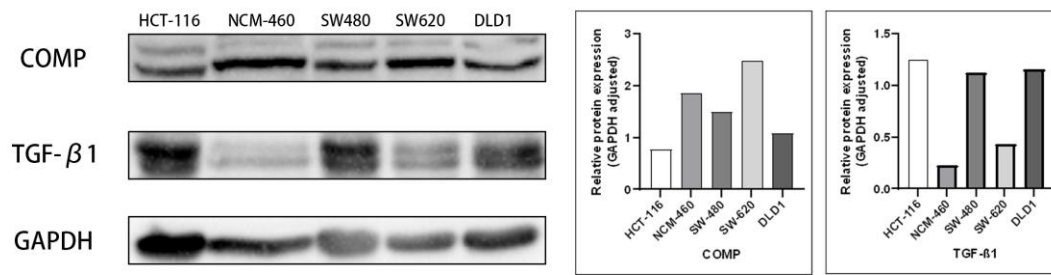

Supplement: Supplementary file 1 [file biomolecules-12-01877-s001.zip › Figure S1.pdf]
